# Reasons for and against participation in studies of medicinal therapies for women with breast cancer: a debate

**DOI:** 10.1186/1471-2288-12-25

**Published:** 2012-03-11

**Authors:** Gero Luschin, Marion Habersack, Irmina-Anna Gerlich

**Affiliations:** 1 Medical University Graz, Auenbruggerplatz 14, 8036 Graz, Austria; 2 Medical University Graz, Gender Medicine & EZA, Graz, Austria

**Keywords:** Breast cancer, Trial participation, Literature, Review

## Abstract

**Background:**

A special challenge for research studies of breast cancer among females is low patient participation rates. We compiled this systematic review to identify reasons why women with, or at high risk of, breast cancer do or do not participate in medicinal studies of breast cancer.

**Method:**

A systematic literature search in the databases Cochrane Library, EMBASE, Medline, Pascal Biomed, ACP Journal Club and CINAHL, as well as searches through reference lists of relevant texts, was performed.

**Results:**

Of 39 relevant full texts, ultimately, nine studies (1 qualitative, 8 quantitative) were included after applying the inclusion criteria. Despite a lack of data material, it was possible to identify various factors influencing women's willingness to participate in medicinal studies and group them into three categories: person-related, study-related, and physician-related.

**Conclusion:**

Reasons for or against participation in studies of medicinal therapies by women with, or at high risk of, breast cancer are multi-dimensional, and should be considered when planning such studies to garner higher participation rates. For a more comprehensive picture of factors that affect participation, further studies in this field are recommended.

## Background

Recruitment of participants in breast cancer trials relative to the incidence of breast cancer patients is somewhat higher than in other cancer types, such as colorectal or lung cancer [1]. However, the number of participants in breast cancer trials is still rather low. Estimates of participation of adult persons in cancer studies is about 1.5-11% of the total number of newly diagnosed or incidental cases [1,2]. Low participation in studies can lead to under-representation, which, in turn, can result in an effect or a clinical effectiveness being shown as not significant [3] or in a failure to obtain theoretical data saturation [4]. Low participation rates in a study may also induce bias, whereby those enrolled do not represent the target population very well. The reason why a planned sample size is not reached within the time frame can, among other things, depend on problems in recruiting participants [5]. Research studies, grouped in reviews, have investigated possible barriers to cancer patients' participation in clinical studies [6-8]. Among these barriers are, for example, randomization, preferences for a certain therapy, degree of knowledge or additional effort in travel [6,7].

In all of these studies however, patients with different cancer types were combined; for example, breast cancer, lung cancer and intestinal cancer, as well as various therapies.

To gain a better understanding of why especially female breast cancer patients frequently do not participate in clinical medicinal studies, a systematic review regarding this sensitive female patient group was conducted.

## Method

### Study type

This review includes both qualitative and quantitative studies that discuss reasons and barriers or influencing factors for the participation of female breast cancer patients in medicinal studies. Clinical medicinal studies in this paper, are defined as studies with a variety of medical therapies, which may include, for example, chemotherapies, endocrine therapies and immune therapies within neo-adjuvant and adjuvant therapy [9].

### Search strategy

In May 2011, a systematic literature search was carried out in the databases: Cochrane, EMBASE, Medline, Pascal Biomed, ACP Journal Club, and CINAHL. The following combination of MeSh-Terms and Keywords was chosen: #1: cancer OR tumo* OR oncolog* OR neoplasms; #2: willing* OR enrol* OR informed consent OR participat*; #3: clinical and trial*; #4: medica* or drug or pharma*; #5: breast; #6: #1 AND #2 AND #3 AND #4 AND #5.

Articles were limited to those published in the last 10 years in German or English. When uncertainties regarding whether the content of the studies was suitable for this review occurred while screening study abstracts (or when articles did not have abstracts), the respective full texts were procured for further evaluation. To find additional relevant literature, the reference lists of the full texts were searched for possible articles.

### Selection criteria

Inclusion criteria: studies that focused on participation or non-participation in clinical medicinal studies of adult women with breast cancer.

Exclusion criteria: studies that did not examine consent-competent female patients, did not focus on medications, investigated the effectiveness of medications, or did not refer to primary sources, study protocols and commentaries.

### Data evaluation

Full texts found in the literature research were evaluated regarding methodical or theoretical rigor by means of the applicable quality checklist for quantitative or qualitative studies of the Alberta Heritage Foundation for Medical Research [10]; texts with values < .75 by these criteria were not used.

### Data analysis

The following data were extracted from the respective full texts: objective, study type/design, method, number and characteristics of the participants, definition of the term "study participation," setting, and results. These extracted data were compared and categorized with regard to factors influencing participation (for and against see Table 1 and 2) in clinical medicinal studies of women, regardless of which research area they originally came from. This categorization was performed according to Mills et al., 2006 [7].

**Table 1:** Reasons for participation in medicinal studies

Reasons for participation	Statistical output
*Altruism (support of medical research and/or helping other patients) *	Support of medical research or helping other people (33%, n = 8) [11]; contribution to therapeutic knowledge (44%, n = 116) [18]; altruism in general (50%, n = 14) [19]; non-participants felt bad due to altruistic reasons as they themselves did not contribute to the study efforts (39%, n = 9) [19]
*Wish for improvement (regarding own chances or regular medical care) *	Improve the chances for breast cancer prevention (*P *= 0.033; OR = 3.16; 95% CI: [1.10-9.06]) [12]; a regular medical care (58%, n = 154) [18], wish for helping themselves (50%, n = 14) [19]
*Concern of breast cancer or often thinking about the topic of breast cancer *	Cancer thoughts (*P *= 0.002; OR = 2.30; 95% CI: [1.40-3.80]) [17], strong concerns regarding breast cancer (*P *= 0.01; OR = 0.15; 95% CI: [0.03-0.77]) [18]
*Physical condition *	Women with non-metastasized carcinoma vs. metastasized carcinoma (RR = 2.80, *P *< 0.01) [14], good-excellent vs. fair-poor health status (*P *= 0.05; OR = 1.47; 95% CI: [1.25-1.98]) [17]
*Age *	49 ± 10 vs. 54 ± 11 years (mean ± SD) (*P *< 0.0001; OR = 1.05; 95% CI: [1.02-1.08]) [17]; under 60 years (*P *= 0.003; OR = 0.40, 95% CI: [0.22-0.73]) [18]
*Memory of close person(s) with breast cancer *	21% (n = 6) valued their own breast cancer risk as low, but nevertheless participated in the study out of respect or a memory of a close person who suffered from breast cancer [19]
*Readiness for treatment in a study *	Nothing to lose by participating (33%; n = 8) [11]; general willingness (60%; n = 160) [18]
*Acceptance of randomization *	Acceptance of randomization (*P *< 0.001; OR = 4.6; 95% CI: [2.7-7.7]) [16]
*No placebo *	Placebo vs. no placebo (RR = 0.80; *P *= 0.05) [14]
*Longer interval from diagnosis/surgery/end of therapy until enrollment*	For non-metastatic trials: if the study protocol planned an interval of 12 weeks or longer between diagnosis/op/end of therapy and recruiting in comparison to a shorter interval (RR = 1.36; *P *< 0.01) [14]
*Already decided once to participate in a medicinal study *	Already having decided to participate in a study (*P *< 0.001; OR = 5.0; 95% CI: [2.9-8.7]) [16]
*Predominantly advantages through the study *	Perceived value of the trial (*P *= 0.020; OR = 2.92; 95% CI: [1.18-7.21]) [12], predominant advantages of the study (50%, n = 14) [19]
*Each treatment strategy seems helpful *	Each of the treatments seems to be beneficial (42%; n = 10) [11]
*Idea of receiving a better treatment *	Idea to receive better treatment (25%; n = 6) [11]
*Feeling, physicians must make decisions *	Medical decision-making preferences (*P *= 0.045; OR = 2.2; 95% CI: [1.0-4.9]) [16]
*Feeling of not being able to reject physician's suggestion *	Unable to refuse the physician's suggestion (*P *= 0.031; OR = 1.8; 95% CI: [1.1-3.2]) [16]
*Satisfaction with receiving information during consultation with physician *	Satisfaction with communication processes (*P *< 0.001; or = 3.1; 95% CI: [1.5-7.8]) [16], satisfaction with the physician's explanations (*P *< 0.001; OR = 9.33; 95% CI: [4.04-21.55]) [18]
*Receiving information regarding financial conflicts of interest*	61-72% (n = 614-724) wish to receive information about financial conflicts of interest [13]. 61-84% (n = 614-845) would participate in a medication study in spite of financial conflicts of interest [13]
*Adequate medical expert knowledge or qualification of the physician *	Clinician expertise and qualifications (*P *= 0.012; OR = 4.9; 95% CI: [1.41-17.04]) [12]

**Table 2:** Reasons against participation in medicinal studies

Reasons for non-participation	Statistical output
*Inconspicuous mammography result *	Inconspicuous result of a mammography (42%, n = 84) [15]
*Additional chronic and/or acute sickness*	Current chronic or acute sickness (20%, n = 40) [15], having a higher risk of developing other diseases than breast cancer (more than 50%, n > 14) [19]
*Skepticism towards clinical studies *	General skepticism towards clinical studies (2%, n = 4) [15]
*Feeling of becoming an "experiment" by participating *	The thought of being an experiment (10%, n = 3) [11]
*Additional family problems or no family support *	Family problems (5%, n = 9) [18]
*Fear of possible side effects*	Side effects (14%, n = 4) [11], (3%, n = 6) [15], 31% (n = 59) [18], 35% (n = 8) [19], willingness decreased from 72% (n = 324) to 52% after explanation of side effects, and to 45% after uterine cancer was mentioned [17]
*Preference of a certain treatment form *	Preferences regarding chemotherapy (21%, n = 6) [11], not the same advantages as a menopausal hormone replacement therapy (22%, n = 5) [19]
*Additional time necessary *	Level of trial inconvenience regarding to the time requirements (*P *= 0.002, OR = 0.10, 95%CI [0.02-0.44]) [12], need too much time for the study (7%, n = 14) [15], additional time and effort needed (22%, n = 5) [19]
*Study lasts too long *	Aversion to taking tamoxifen for 5 years (14%, n = 4) [11], study duration (6%, n = 11) [18]
*Too far to travel from home to place of study *	Too far to travel from home to the examination center (12%, n = 24) [15], distance (4%, n = 8) [18]
*Randomization *	Aversion regarding the allotment of treatments in the study (38%, n = 11) or regarding the randomization (17%, n = 5) [11], randomization (39%, n = 9) [19]
*Fear of medication abuse *	Medication abuse (33%, n = 64) [18]
*Incompatibility of own therapy and study medication *	Incompatibility of their hormone replacement therapy with the study medication (22%, n = 5) [19]
*Concern of not receiving appropriate therapy for oneself *	Concern of receiving the appropriate treatment (7%, n = 2) [11]
*Not willing to lose control over personal decisions *	Loss of control (7%, n = 2) [11], women who refused showed preferences regarding personal decision making (72%) versus women who accepted (35%) (*P *< 0.001) [16]
*Not willing to decide for oneself regarding participation *	Not want to make own decision because physician should decide (10%, n = 3) [11]
*Physician's advice not to participate *	Physician counsel not to participate (24%, n = 46) [18]

## Results

**Figure 1:**
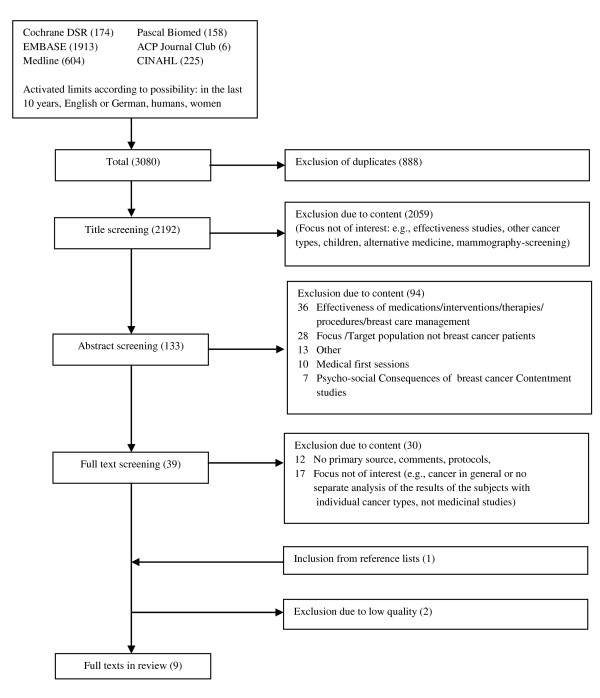
**Flow chart of study selection process**.

After applying the selection criteria according to the title and abstract screening, 39 publications (of initially 3080 references) remained for further evaluation. Twelve publications were excluded as secondary literature, comments or protocols; 17 publications were excluded because their focus was not of interest (e.g., medicinal studies). One additional article was included from the reference lists. Two articles had to be excluded as they each had a quality score of < 0.75. Ultimately, 9 publications met the inclusion criteria and were subjected to further analysis and evaluation (see Figure 1).

Eight of these nine included studies were quantitative [11-18]; only one was qualitative [19]. The summarized data extraction of the individual studies is found in Table 3. The quality assessments performed, including comments, are depicted in Tables 4 and 5, according to research areas. Four of the studies were conducted in the United States [12,13,17,19], two in Canada [11,14], and the rest in Europe; in Germany [15], France [16] and Italy [18]. All of these articles have been published in English.

**Table 3:** Methodological descriptions of the studies (N = 9)

Reference	Country	Objective/Question	Design	Method	Participants	Data analysis
*Altschuler & Somkin 2005 *[19]	USA	Why did women who initially showed interest in participating in the STAR trial make different decisions about whether to participate or not participate? (tamoxifen or raloxifene)	Qualitative chemo-prevention study	Half-structured deep interviews	51 post-menopausal women with high breast cancer risk(28 participants and 23 non-participants)	Grounded theory
*Ellis et al 2002 *[11]	Canada	Evaluation of information brochure, regarding theoretical willingness to participate in a 6-month clinical trial for breast cancer treatment (Tamoxifen alone, chemotherapy alone or combined)	Quantitative before-after-study	Questionnaires closed answers	Before: 83 women with early invasive breast cancer After: 67 women	Descriptive statistics
*Houlihan et al 2010 *[12]	USA	Investigation of factors influencing women's decision to participate in breast cancer prevention trial (tamoxifen, raloxifene).	Quantitative case-control study	Questionnaires through mail	Of 242 post-menopausal women, 81 participated	Logistic regression model
*Kim et al 2004 *[13]	USA	When financial conflicts of interest were stated on consent forms, which respondents refused to participate in a study using a new medication solely on the basis of these conflicts of interest?	Quantitative study	Internet questionnaires; closed answers	1006 female breast cancer patients	Descriptive statistics
*Lemieux et al 2008 *[14]	Canada	Identifying barriers in the study protocols, with respect to the low recruitment rates in clinical breast cancer trials, 1997-2002.	Quantitative study	Questionnaires; closed answers	616 women participated in studies of Phase III (or II+ III) and 72 in studies of Phase II (or I+II).	Multivariate analysis
*Loehberg et al 2010 *[15]	Germany	Identification of characteristics of women who wanted to receive information about a Phase II chemoprevention study.	Quantitative, multi-centered study	Questionnaires	202 women of 446 wanted to receive further information about the risk of breast cancer; 3 women decided to participate.	Descriptive statistics
*Mancini et al 2007 *[16]	France	Identification of preferences in decision-making processes in relation to participation in another clinical drug trial.	Quantitative, prospective cohort study	Questionnaires	Of 455 women 267 were invited to the study; of these 201 agreed to participate and 66 declined.	Descriptive statistics and multivariate analysis
*Mandelblatt et al 2005 *[17]	USA	Effectiveness of a brief consultation and an informational brochure compared with use of brochure only in recruitment for a breast cancer prevention study. (tamoxifen and raloxifene)	Quantitative, simple randomized controlled study	Questionnaires	232 of 450 women participated in consultation +brochure-group; 218 of 450 in the brochure-only-group	Descriptive statistics and logistical regression model
*Rondanina et al 2008 *[18]	Italy	Socio-demographic, health-related and psychological factors influencing decision to participate or not in a five-year hormone replacement therapy. (HRT-Phase III, low-dose tamoxifen)	Quantitative study	Questionnaires through mail	496 of 1457 women participated in the HET-study	Descriptive statistics and multivariate analysis

**Table 4:** Quality assessment according to AHFMR 2004 (N = 1)

Criteria	Assessment*	Remarks
*Altschuler & Somkin 2005 *[19] (Score = 0.80**)		
Research question(s)/Objective(s)	Yes	Described clearly and evident throughout the text
Study design	Partially	Not explicitly named; inconsistencies in subsequent data collection not evident
Study context	Yes	Setting is described
(Theoretical) frame of reference	Partially	Study objective does not completely follow the knowledge that is depicted in the introduction
Sampling	Partially	Described and reproducible; random sampling was performed until theoretical data saturation was reached; consent of women was obtained before the interviews began. Duration of study not mentioned.
Data collection	Yes	Reproducible
Data analysis	Yes	Categories, codes, and memos described; categories listed explicitly
Reliability	Yes	Second author verified formation of categories/assignment of codes by the first author; if there were discrepancies in the codes, they went back to the original material and found consensus
Conclusions	Yes	Results discussed; results of other studies are drawn upon as comparison
Reflexion	Partially	Possible influence of financial compensation were not reflected; recall bias listed as possible weakness of the study

*Possible categories: yes, partially, no, not applicable

** Ascertainment according to AHFMR 2004 (p. 20) = ((number "yes" * 2) + (number "partially" * 1))/20

**Table 5:** Quality assessment according to AHFMR 2004 (N = 8)

Criteria	Assessment*	Remarks
*Ellis et al 2002 *[11] (Score = 0.71**)		
Research question(s)/Objective(s)	Yes	At the beginning of the introduction; contains dependent/independent variables, including population
Study design	Partially	Not explicitly described, no inconsistencies in the subsequent data collection
Sampling	Yes	Described, exclusion criteria mentioned; written consent obtained in each case
Sample characteristics	Yes	Basic information given and depicted in tables
Randomization	Partially	Randomization performed; exact process of randomization not described
Blinding: Researchers	n.a.	Not possible
Blinding: Participants	n.a.	Not possible
Data collection	Partially	Categories of questionnaires given
Sample size	Partially	Power analysis performed; no information about low response rate of second questionnaire
Data analysis	Partially	Regression appropriate; no indication regarding the characteristics of participating/non-participating women
Variance estimate	Yes	Confidence intervals and ranges indicated
Confounding factor control	Partially	Control at analysis level with multivariate model, but no attempt to standardize the physician's consultation
Result depiction	Partially	Described in the text; the secondary result (change of knowledge) was not depicted graphically/in a table.
Conclusions	Yes	Relevant results are discussed and compared with other studies
*Houlihan et al 2010 *[12] (Score = 0.86**)		
Research question(s)/Objective(s)	Yes	Listed
Study design	Yes	Stated
Sampling	Partially	Inclusion criteria mentioned, procedure not described
Sample characteristics	Partially	Information only regarding city and ethnic group given; average age of the women only in Discussion section (but without standard deviation); other basic data are not available
Randomization	n.a.	Not possible
Blinding: Researchers	n.a.	Not possible
Blinding: Participants	n.a.	Not possible
Data collection	Partially	Reproducible on a limited basis
Sample size	Yes	No power or variance analysis given; no problems with multiple tests described and significant values were obtained
Data analysis	Yes	Described
Variance estimate	Yes	Confidence intervals indicated
Confounding factor control	Yes	Control at analysis level with multivariate model
Result depiction	Yes	Described; significant results depicted in tables
Conclusions	Yes	Results depicted in a summarized manner and discussed with previous studies; limitations and recall bias reflected
*Kim et al 2004 *[13] (Score = 0.75**)		
Research question(s)/Objective(s)	Yes	Clearly formulated and discussed throughout the text
Study design	Partially	That this is a non-comparative study only becomes obvious in the results section
Sampling	Partially	Described; random- but convenience sample
Sample characteristics	Partially	Basic data exist, but not tested regarding differences between the sub-groups
Randomization	Partially	Would have been possible
Blinding: Researchers	n.a.	Not possible
Blinding: Participants	n.a.	Not possible
Data collection	Yes	Reproducible
Sample size	Yes	Variance analysis; significant values obtained
Data analysis	Partially	Scenarios of conflicts of interest are listed; the questions/answer options/data analysis procedure described, results in the text do not match the table data
Variance estimate	Yes	Variance estimate performed
Confounding factor control	Yes	Control at analysis level with multivariate model
Result depiction	Yes	Results summarized and described in tables
Conclusions	Partially	Results discussed and compared with other studies; limits listed, possible influence on the results through the chance of winning 3 × $500 was not reflected
*Lemieux et al 2008 *[14] (Score = 0.82**)		
Research question(s)/Objective(s)	Yes	At the beginning of the method section; contains variables to examine, population, place and timeframe
Study design	Partially	Not explicitly mentioned, but no inconsistencies result in the subsequent data collection
Sampling	Partially	Procedure described, exclusion criteria listed; but bias possible as the selection of cooperatives and pharmaceutical companies was made by experts
Sample characteristics	Yes	Basic information given, steps in the categorization mentioned
Randomization	n.a.	Not possible as the authors wanted to include all studies in Ontario from the years 1999-2002
Blinding: Researchers	n.a.	Not possible
Blinding: Participants	n.a.	Not possible
Data collection	Yes	Reproducible
Sample size	Partially	No power analysis
Data analysis	Yes	Poisson Regression appropriate; handling of missing values described
Variance estimate	Yes	Confidence intervals and ranges stated
Confounding factor control	Partially	Control at analysis level with multivariate model, but the institutions received money as incentive to participate in the study
Result depiction	Yes	Results depicted in the text and tables
Conclusions	Yes	Results discussed and compared with other studies
*Loehberg et al 2010 *[15] (Score = 0.77**)		
Research question(s)/Objective(s)	Partially	At the end of the introduction; calculation of influencing factors through multiple regression analyses unclear
Study design	Partially	Not explicitly mentioned, no inconsistencies
Sampling	Yes	Procedure described, inclusion criteria mentioned
Sample characteristics	Yes	Basic information given
Randomization	n.a.	Not possible
Blinding: Researchers	n.a.	Not possible
Blinding: Participants	n.a.	Not possible
Data collection	Partially	Reproducible
Sample size	Yes	No power- or variance analysis given; no problems mentioned with multiple tests
Data analysis	Yes	Described
Variance estimate	Yes	Confidence intervals and distribution indicated
Confounding factor control	Yes	Analysis of sub-groups conducted
Result depiction	Partially	Results depicted in the text and tables do not all follow the objective of the article, but seem to be appropriate in general.
Conclusions	Partially	Results discussed and compared with other studies; no critical reflection that the information could possibly influence the number of participants
*Mancini et al 2007 *[16] (Score = 1.00**)		
Research question(s)/Objective(s)	Yes	Described precisely
Study design	Yes	Described and appropriate
Sampling	Yes	Procedure described; inclusion criteria mentioned; consent forms collected
Sample characteristics	Yes	Basic information on the women and sub-groups given;
Randomization	n.a.	Not possible
Blinding: Researchers	n.a.	Not possible
Blinding: Participants	n.a.	Not possible
Data collection	Yes	Reproducible
Sample size	Yes	No power- or variance analysis, sample size seems to be sufficiently large
Data analysis	Yes	Logistical regression analysis appropriate, individual tests within the framework of descriptive statistics conducted; significance level mentioned
Variance estimate	Yes	Confidence intervals and standard deviations given
Confounding factor control	Yes	Control at analysis level with multivariate model
Result depiction	Yes	Results of regression analysis are listed in the text and table; not all significant results of the comparisons within the framework of descriptive statistics were also described in the text, but no inconsistencies result
Conclusions	Yes	Results discussed and compared with other studies
*Mandelblatt et al 2005 *[17] (Score = 0.79**)		
Research question(s)/Objective(s)	Partially	Described in the abstract, formulation of objective in the text fails to mention the investigation of two interventions
Study design	Yes	Described and appropriate
Sampling	Yes	Procedure described; inclusion criteria mentioned; consent forms collected
Sample characteristics	Yes	Basic information on the women and sub-groups given
Randomization	Yes	Randomization performed; procedure described
Blinding: Researchers	n.a.	Not possible
Blinding: Participants	n.a.	Not possible
Data collection	Yes	Reproducible
Sample size	Yes	Not obvious whether a power analysis was conducted later; the sample size seems to be sufficient
Data analysis	Partially	Logistical regression analysis appropriate, tests conducted within the framework of descriptive statistic analysis are not mentioned; handling of missing values described; significance level not given
Variance estimate	Yes	Confidence intervals, standard deviations and range given
Confounding factor control	Partially	Control at analysis level with multivariate model; but in asking the control group, the same standardized questionnaire as in the intervention group was used
Result depiction	Partially	Secondary results depicted in the text and tables; but the primary result is not mentioned in the text, it is only listed in the table; recording of influencing factors not conducted for both interventions. Differences regarding the objective breast cancer risk between those women who consented to participation in the medication study and those who refused participation were not pointed out. The text only lists percentages and no absolute numbers; consequently, readers have to infer from the tables how many women in total participated in the data collection, and subsequently in the medication study.
Conclusions	Partially	Results discussed but only partially compared with other studies
*Randonina et al 2008 *[18] (Score = 0.91**)		
Research question(s)/Objective(s)	Yes	Appears in the middle of the method section, primary and secondary results are mentioned at the end of the introduction
Study design	Partially	Not mentioned explicitly for this collection process, only the design of the medication study is described
Sampling	Yes	Procedure described; including criteria mentioned; consent forms collected
Sample characteristics	Yes	Basic information on the women and sub-groups given
Randomization	n.a.	Not possible
Blinding: Researchers	n.a.	Not possible
Blinding: Participants	n.a.	Not possible
Data collection	Yes	Reproducible
Sample size	Yes	Power analysis given
Data analysis	Yes	Analysis appropriate and apparent from the objective; handling of missing values described; significance level given
Variance estimate	Yes	Confidence intervals, standard deviations and ranges given
Confounding factor control	Yes	Control at analysis level with multivariate model; also, control regarding age distribution based on low response rate for questionnaires; furthermore, an attempt was made to standardize the preceding consultation session
Result depiction	Partially	Results depicted in the text and tables and correspond with each other; but results of the regression analysis are interpreted in the text as "in connection with" and not as "influence" - the interpretation in the framework of the remark in the table is again depicted correctly
Conclusions	Yes	Results discussed and compared with other studies

* Possible categories: yes, partially, no, not applicable

** Ascertainment according to AHFMR 2004 (p. 14) = ((number "yes" * 2) + (number "partially"" * 1))/(28 - (number "n.a." *2))

In the articles, a great variety of study designs were utilized as case-control [12], cohort [16] or randomized studies [17] (among others), where the design was not explicitly identified in most of them [11,13-15,18,19]. The prevalent method used to elicit women's reasons for or against participation in studies with medicinal therapies were questionnaires [11-13,15-18]. Most of the articles referred to investigations already performed, to identify the relevant reasons for consent or refusal to participate [12,14,15,18,19]. Three studies, though, used theoretical scenarios for the solicitation of reasons [11,13,16]. In one article, on the other hand, women were asked regarding their intent to participate in a currently active study [17].

The majority of the studies focused on chemotherapies [11,12,15-17,19], whereas only one particularly referred to endocrine therapy [18], one to a new aromatase inhibitor, other endocrine therapy and chemotherapy [14], and another one generally to medicines [13].

Half of the studies addressed post-menopausal women, each with varying degrees of breast cancer risk [12,15,17-19], and half of the studies addressed women with invasive breast cancer [11,13,14,16]. The studies mainly investigated women with an average age of 53 (range: 40-66 years) [11-18]. One study mainly included women in age groups 50-59, 60-69 and 70-79 years [19].

## Discussion and conclusions

The various factors influencing participation in breast cancer medicinal research identified in the nine studies were placed by the authors into three categories: person-related, physician-related and study-related.

The person-related category comprised health, psycho-social and demographic reasons. A younger age (demographic reason) was identified as a factor influencing willingness to participate [15,17,18].

Regardless of whether study participants were younger or older, they frequently had high subjective perceptions of their risk of breast cancer [16,18], although their objective risks, assessed according to Gail scores, in one of the two studies, were relatively low on average [17,19,20]. Another study also showed women who had participated in medication studies assessed their breast cancer risk subjectively much higher than women who had not participated, though both groups did not differ according to their Gail scores [19]. This suggests that, in future studies, subjective perceptions of risk should be addressed. Also, in ovarian cancer investigations, potential subjects' higher personal risk perception and concern raised the probability of making use of screening [21,22]. These results suggest that women's participation in such studies depends more on subjective risk than objective risk. Though subjective breast cancer risk in two studies in this review correlated with the women's willingness to participate [17,19], in another medication study, women's participation was more likely when they were less personally concerned about breast cancer [18]; however, this last-mentioned study does not reveal whether relatives of the study participants had suffered from breast cancer or not, which could increase the subjects' concern, and thus interest, in participating in a medication study. One study showed women who had first and second degree relatives with breast cancer requested information about the medication study twice as often as women who did not have this diagnosis among their relatives (OR 2.35, 95% CI, 0.99-5.57)[15].

Studies show a negative correlation between the concern regarding breast cancer and satisfaction with the physician's consultation [23]. Nevertheless, satisfaction with physicians' consultation and communication processes (physician-related reason) was mentioned as reason for participation/non-participation in medication studies [16,18], indicating that patient-clinician relationships play a decisive role in patients' willingness to participate in a study.

Identification of potential differences in consultations in the medication studies was not possible [11,13,16] because only one study reported the content of the conversations [17]. Therefore, not all women in the studies might have been informed about the same things. To avoid this distortion in future studies, the use and documentation of conversation manuals in these consultations seems advisable.

The main study protocol-related reason against participation was additional time needed [12,18,19].

The randomization procedure was mentioned in two studies as reason against participation [11,19]; willingness for randomization was mentioned in one study as an influencing factor for participation [16]. In cancer research, lack of understanding of the principle of randomization has been researched as barrier to subject participation [24,25]. Another study showed that, among those who initially decided against participation in randomized studies, more than half ultimately consented to participation after they had received more detailed information regarding the randomization process [26]. Though this connection was not identifiable from studies in this review, our results showed that randomization could influence participation in medication studies. Informing potential study subjects of the reasons for randomization could therefore promote their participation.

Fear of possible side-effects (a treatment-related reason) was also frequently mentioned as reason for non-participation [11,15,18,19], suggesting that the probabilities for possible side-effects should be explained extensively during recruitment.

The review shows that the willingness to participate in the theoretical scenarios was considerably higher (58%; range: 25-75%) [11,13,16] than in studies that were actually, or yet to be, conducted (27%; range: 1.5-55%) [12,14,15,17-19]. High willingness to participate in hypothetical scenarios is also seen in other studies [26-28]. Two studies collected their data retrospectively [12,19], with the risk of recall bias in the results. Two other studies counteracted this bias by collecting would-be participants' relevant reasons immediately after consent or rejection of participating in their respective study [15,18]. This procedure could also prevent such distortion in future studies.

A limitation of this review concerns its ten-year time frame. Although more full texts might have been included if our criteria allowed older investigations, the primary goal of this review was to identify *current *studies; we therefore restricted this study to the past decade.

The strengths of this review include its use of an extensive assessment scheme, allowing comprehensive quality evaluation of the respective articles, using consistent criteria. This scheme also could function as a kind of checklist, thus reducing the probability of forgetting any items in the assessment. Another strong point was that six databases were searched, allowing wide coverage of possible publications, as a result, of the articles gleaned from the references of all the full texts, only one was found that had not been part of the original database literature research. Inclusion of only high-quality studies is a further strength, as all studies had to show a high quality, of at least 75%, to be included in this review. Two studies did not meet this requirement and were therefore not included in the results [29,30].

Physicians' viewpoints as to why women with breast cancer or breast cancer risk choose or decline to participate in medication studies is being researched, both with regard to general cancers [31] and breast cancer [24,32], but not specifically with medication studies.

In sum, this review shows that the reasons for participation/non-participation in medication studies are multifactorial. Moreover, while factors affecting patient participation in medication studies are obviously useful to know in planning and realizing future investigations, few such insights are currently available, apparently due to the small number of relevant studies; further quantitative and qualitative research is needed.

## Competing interests

The authors declare that they have no competing interests.

## Authors' contributions

All authors carried out the literature research, evaluated texts separately and discussed the results together. All authors drafted the manuscript and approved the final version.

## Pre-publication history

The pre-publication history for this paper can be accessed here:

http://www.biomedcentral.com/1471-2288/12/25/prepub
